# Supplementary figures and images for: Molecular characterization, expression patterns and cellular localization of BCAS2 gene in male Hezuo pig
Source: PeerJ. 2023 Oct 24;11:e16341. doi: 10.7717/peerj.16341 (PMC10607209; doi:10.7717/peerj.16341)

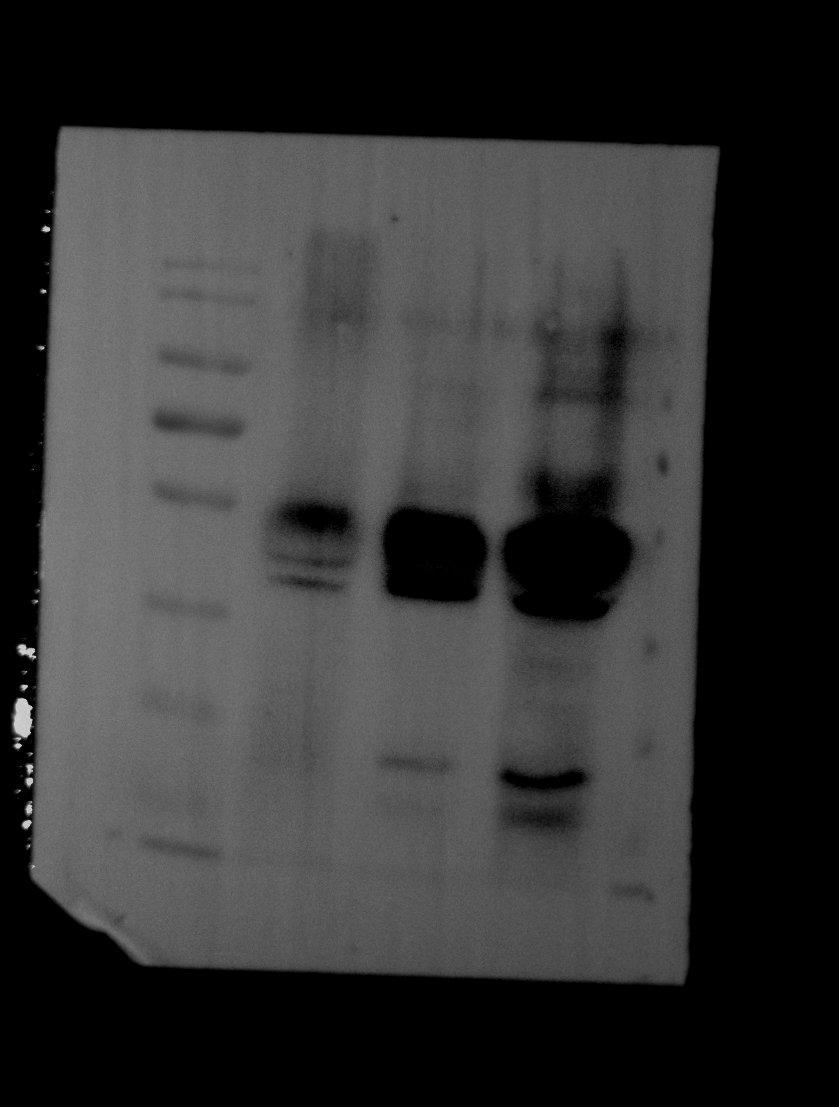

Supplement: Supplemental Information 4 [file peerj-11-16341-s004.zip › full-length uncropped gels/Figure 6C-original1-BCAS2.tif]

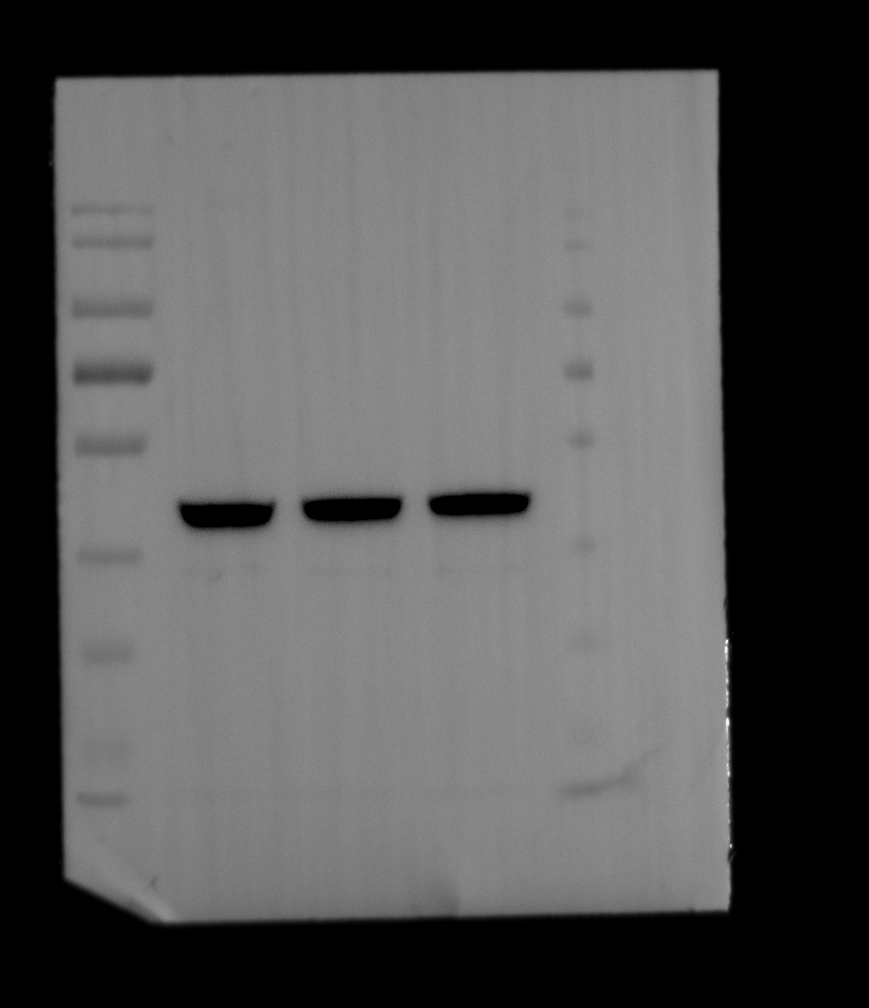

Supplement: Supplemental Information 4 [file peerj-11-16341-s004.zip › full-length uncropped gels/Figure 6C-original1-a┬-actin.tif]

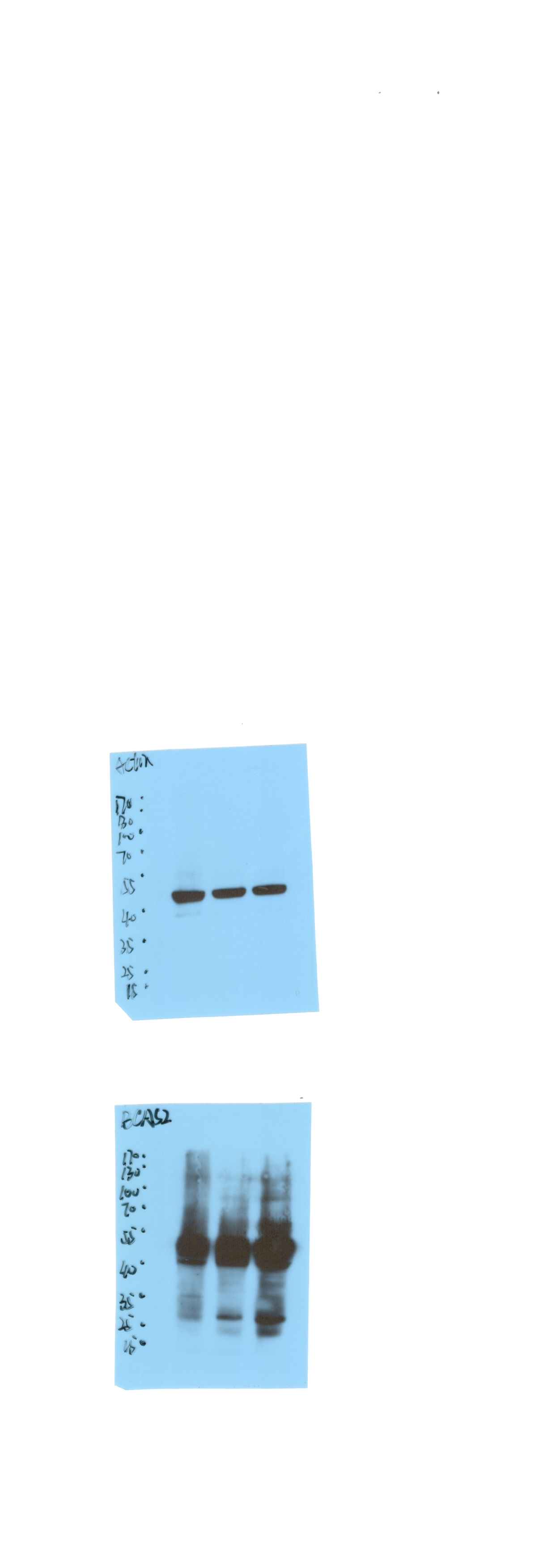

Supplement: Supplemental Information 4 [file peerj-11-16341-s004.zip › full-length uncropped gels/Figure 6C-original2.tif]

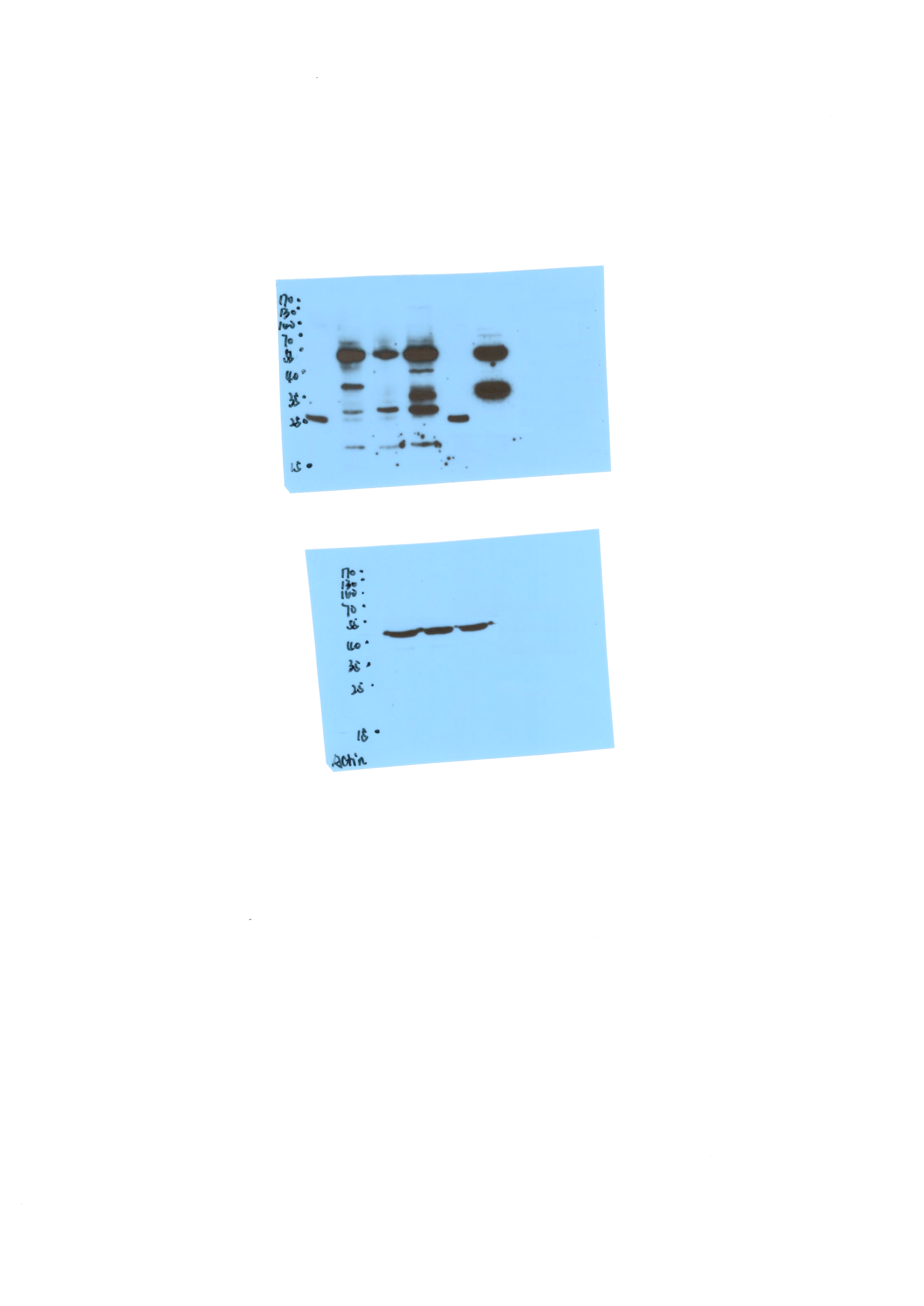

Supplement: Supplemental Information 4 [file peerj-11-16341-s004.zip › full-length uncropped gels/Figure 6C-original3.tif]

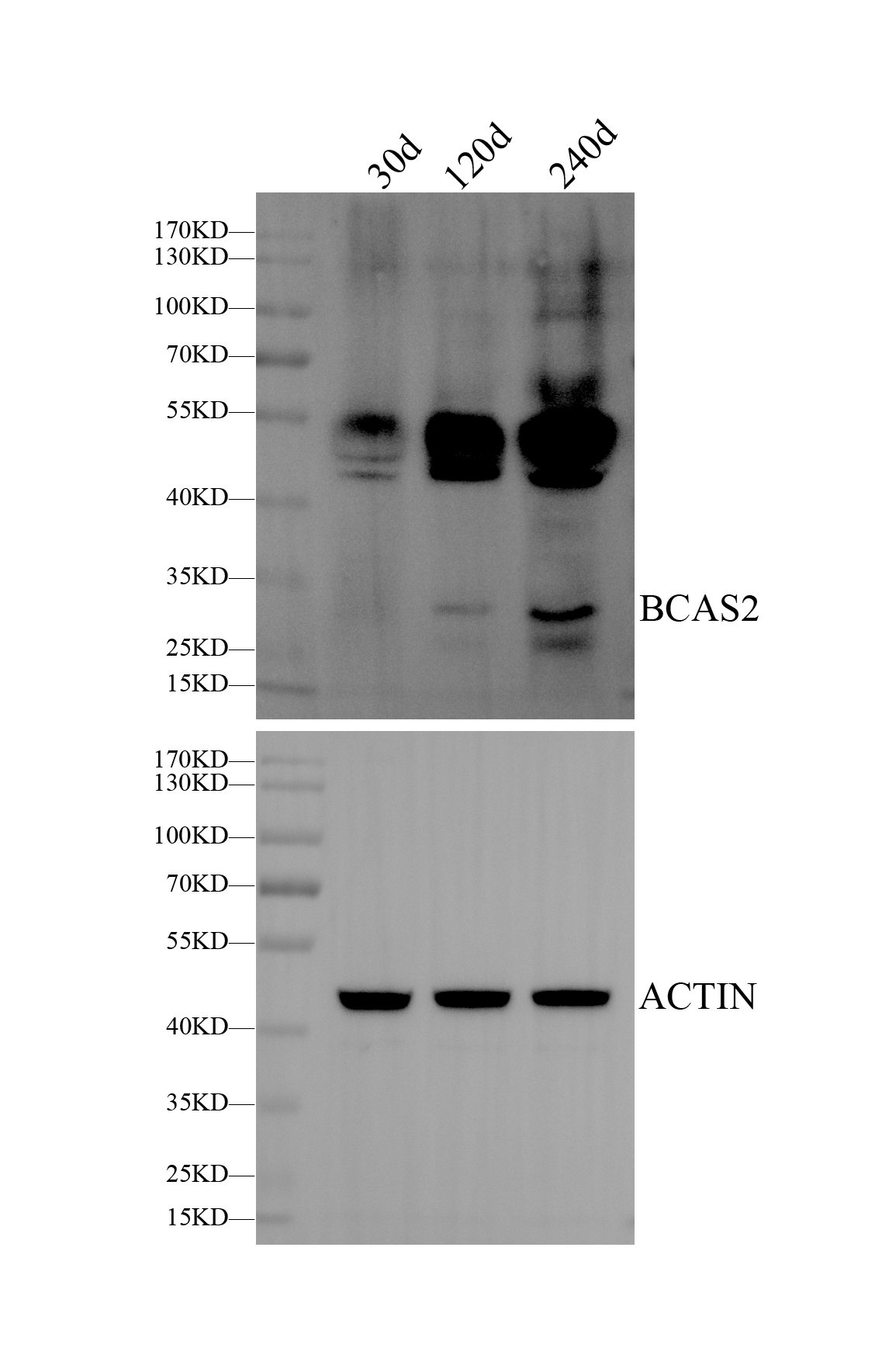

Supplement: Supplemental Information 4 [file peerj-11-16341-s004.zip › full-length uncropped gels/Figure 6C-tidy1.tif]

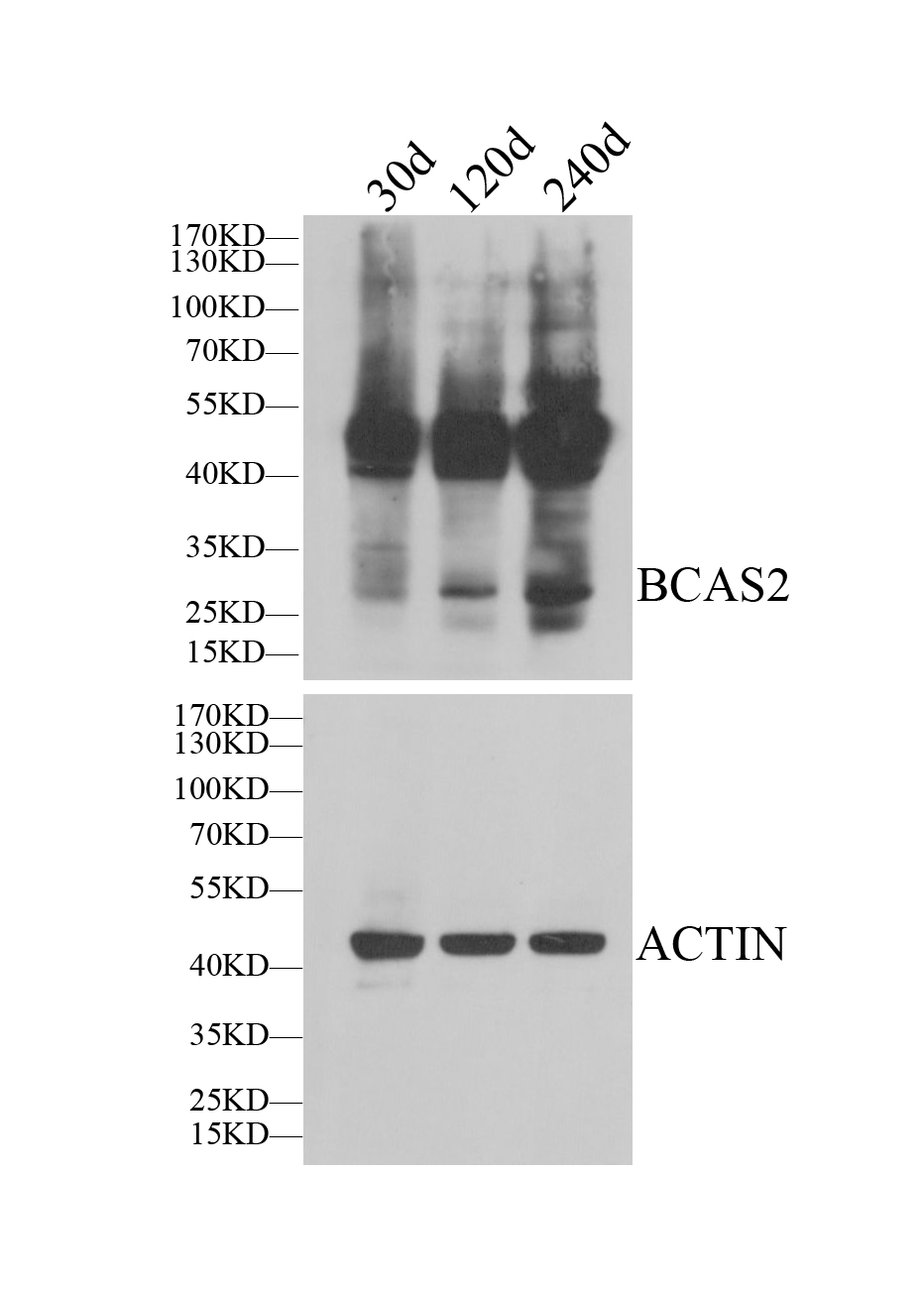

Supplement: Supplemental Information 4 [file peerj-11-16341-s004.zip › full-length uncropped gels/Figure 6C-tidy2-grey.tif]

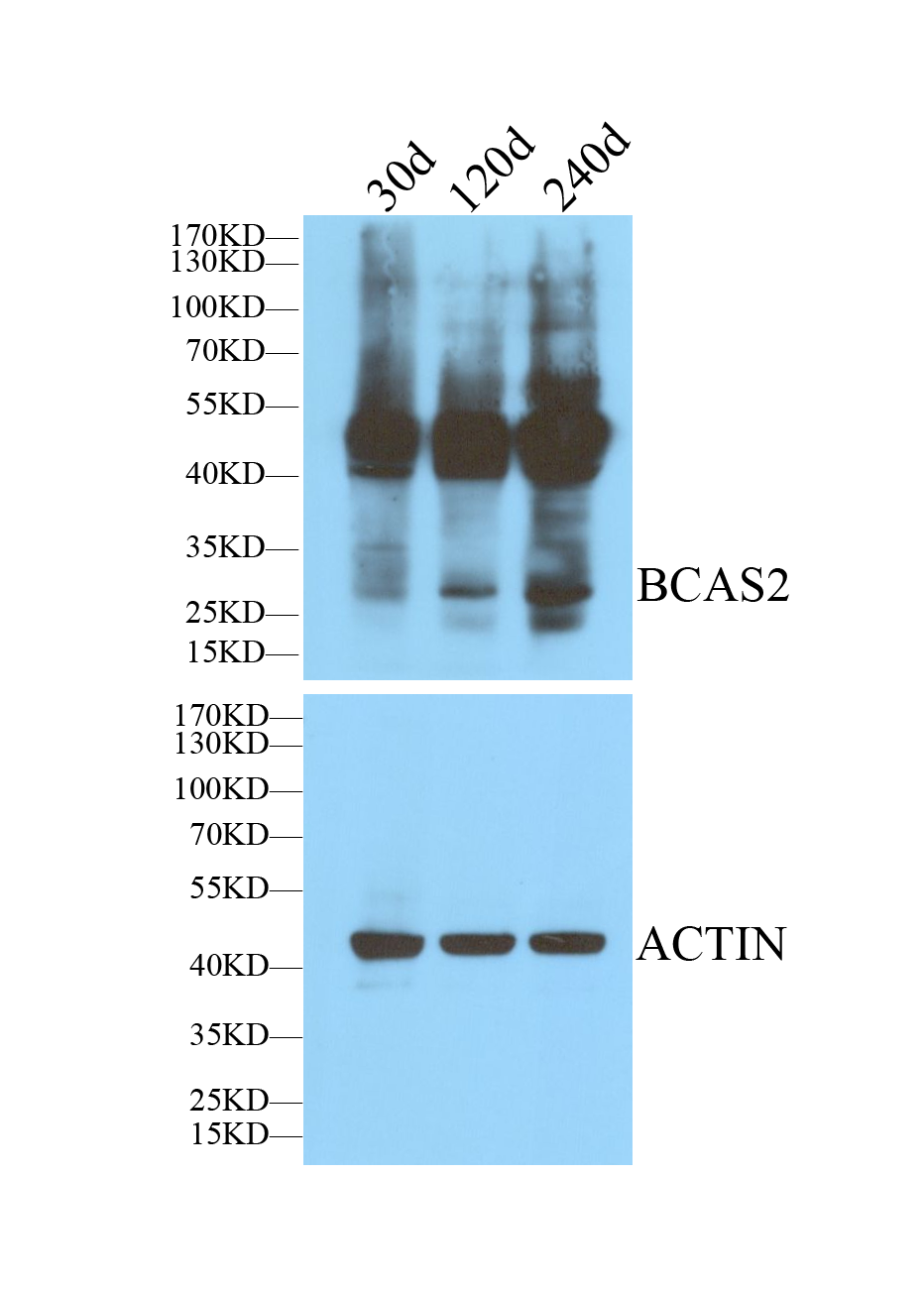

Supplement: Supplemental Information 4 [file peerj-11-16341-s004.zip › full-length uncropped gels/Figure 6C-tidy2.tif]

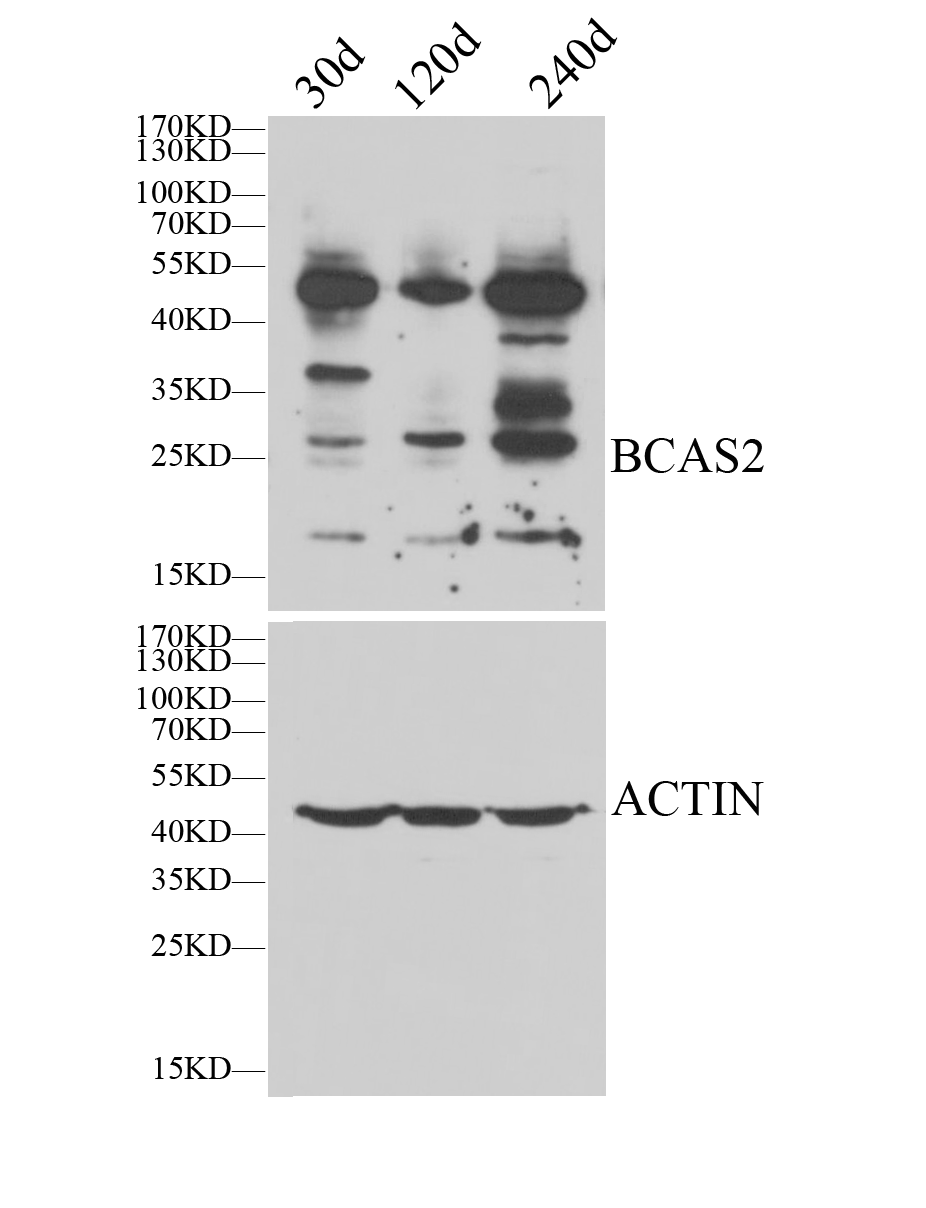

Supplement: Supplemental Information 4 [file peerj-11-16341-s004.zip › full-length uncropped gels/Figure 6C-tidy3-grey.tif]

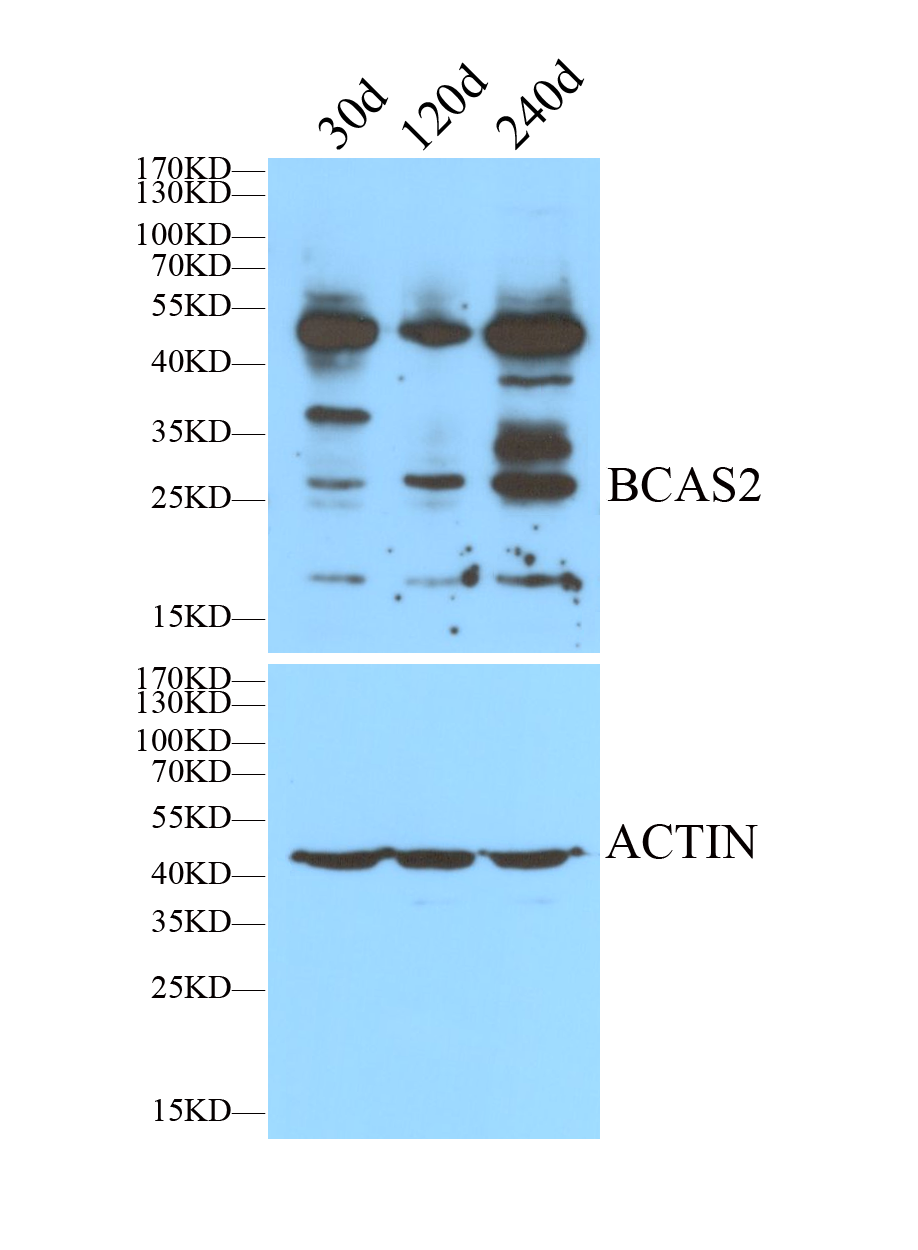

Supplement: Supplemental Information 4 [file peerj-11-16341-s004.zip › full-length uncropped gels/Figure 6C-tidy3.tif]

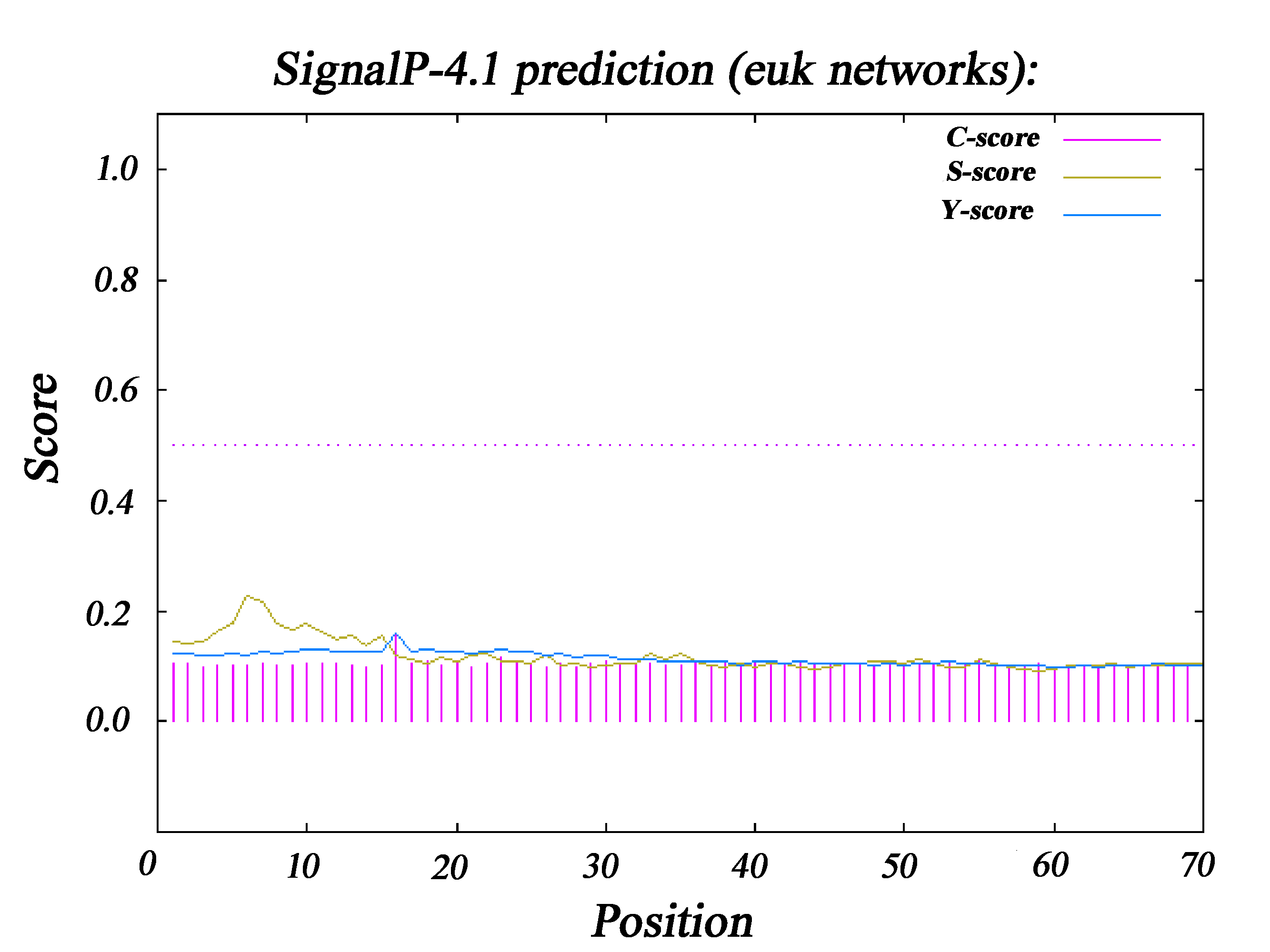

Supplement: Supplemental Information 5 [file peerj-11-16341-s005.png]

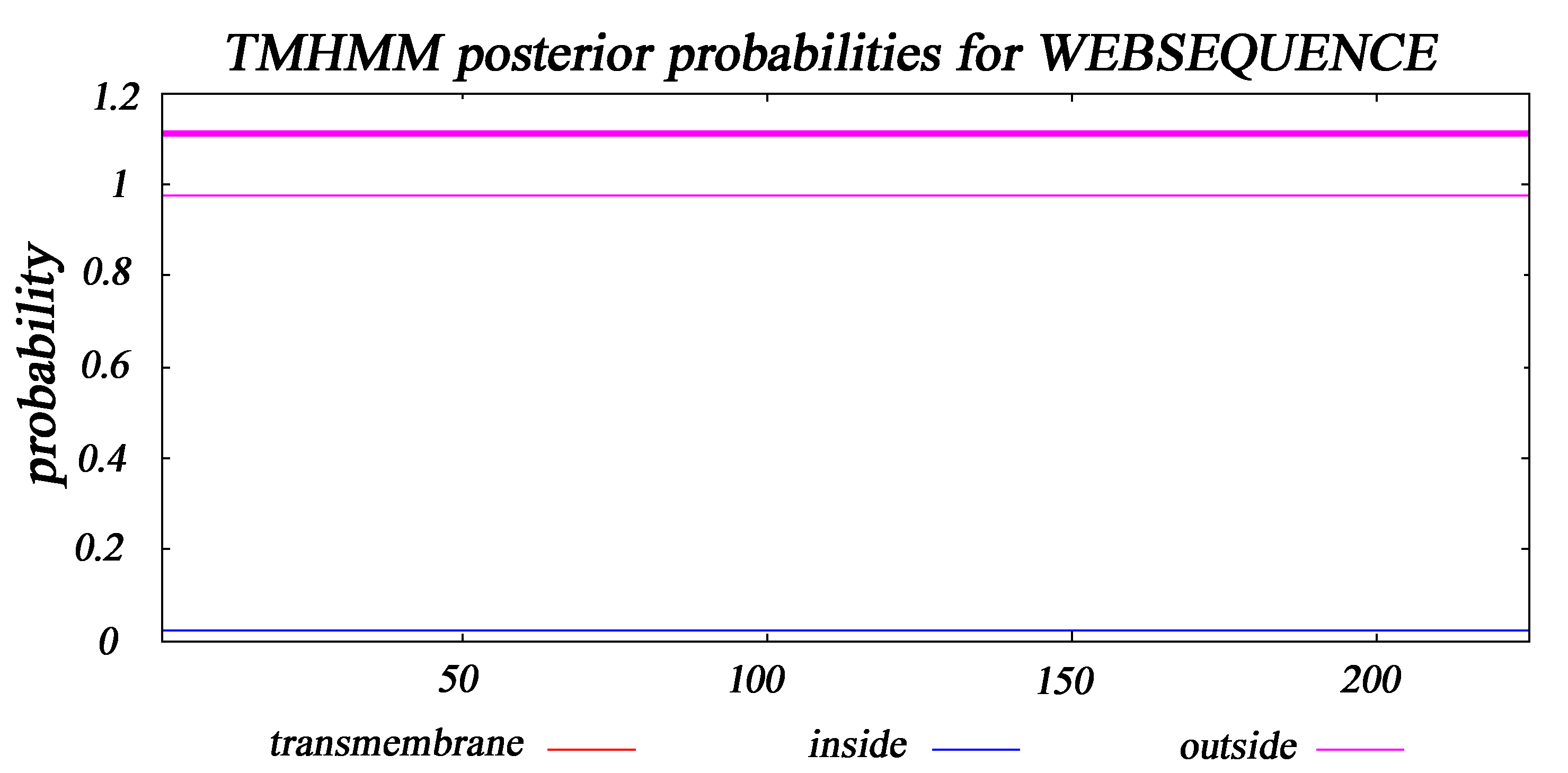

Supplement: Supplemental Information 6 [file peerj-11-16341-s006.png]
