# Supplementary material for: Molecular characterization, expression patterns and cellular localization of BCAS2 gene in male Hezuo pig
Source: PeerJ. 2023 Oct 24;11:e16341. doi: 10.7717/peerj.16341 (PMC10607209; doi:10.7717/peerj.16341)
Supplement: Supplemental Information 7 [file peerj-11-16341-s007.docx]

**Table S1. Alignment of the similarity of nucleotide and amino acid sequence of *BCAS2* CDS region between Hezuo Pig and other species.**

| **Species** | **Genbank No.** | **Nucleotide similarity %** | **Amino acid similarity %** |
| --- | --- | --- | --- |
| *Sus scrofa* (pig) | NM_001244424.1 | 99.56 | 100 |
| *Camelus dromedarius* (Arabian camel) | XM_031457863.1 | 95.28 | 100 |
| *Camelus bactrianus* (Bactrian camel) | XM_010968385.2 | 95.28 | 100 |
| *Vicugna pacos* (alpaca) | XM_015247483.2 | 94.99 | 100 |
| *Equus caballus* (horse) | XM_001499911.6 | 94.40 | 98.23 |
| *Canis lupus familiaris* (dog) | XM_038691841.1 | 94.01 | 99.49 |
| *Ovis aries* (sheep) | XM_027973457.2 | 93.81 | 100 |
| *Capra hircus* (goat) | XM_018045943.1 | 93.81 | 100 |
| *Felis catus* (domestic cat) | XM_004001122.6 | 93.79 | 99.56 |
| *Macaca nemestrina* (pig-tailed macaque) | XM_011736984.1 | 93.66 | 98.23 |
| *Bos taurus* (cattle) | NM_001015540.1 | 93.51 | 100 |
| *Bos indicus* (zebu cattle) | XM_019956763.1 | 93.36 | 100 |
| *Colobus angolensis palliates* | XM_011933114.1 | 93.36 | 98.23 |
| *Mandrillus leucophaeus* (drill) | XM_012000793.1 | 93.22 | 98.23 |
| *Bubalus bubalis* (water buffalo) | XM_006064812.3 | 93.22 | 100 |
| *Canis lupus dingo* (dingo) | XM_025453447.2 | 93.20 | 99.56 |
| *Homo sapiens* (human) | NM_005872.3 | 93.07 | 98.23 |
| *Rattus norvegicus* (Norway rat) | NM_001106458.2 | 92.92 | 98.23 |
| *Oryctolagus cuniculus* (rabbit) | XM_002715751.3 | 92.04 | 98.23 |
| *Odocoileus virginianus texanus* | XM_020875874.1 | 91.76 | 100 |
| *Panthera pardus* (leopard) | XM_019457924.1 | 91.29 | 92.04 |
| *Mesocricetus auratus* (golden hamster) | XM_013118129.3 | 90.18 | 99.45 |
| *Mus musculus* (house mouse) | NM_001356330.1 | 87.80 | 90.71 |
| *Pan troglodytes* (chimpanzee) | XM_513670.4 | 87.46 | 97.35 |
